# Impact of adenomyosis and endometriosis on IVF/ICSI pregnancy outcome in patients undergoing gonadotropin-releasing hormone agonist treatment and frozen embryo transfer

**DOI:** 10.1038/s41598-023-34045-7

**Published:** 2023-04-25

**Authors:** Yu Wang, Yu-Chiao Yi, Hwa-Fen Guu, Ya-Fang Chen, Hsiao-Fan Kung, Jui-Chun Chang, Li-Yu Chen, Shih-Ting Chuan, Ming-Jer Chen

**Affiliations:** 1 Division of Reproductive Endocrinology and Infertility, Department of Obstetrics, Gynecology and Women’s Health, Taichung Veterans General Hospital, 1650 Taiwan Boulevard Sect. 4, Taichung, 40705 Taiwan; 2 Department of Obstetrics and Gynecology, School of Medicine, National Yang Ming Chiao Tung University, No.155, Sec.2, Linong St. Beitou Dist., Taipei City, 112304 Taiwan

**Keywords:** Diseases, Endocrinology, Medical research

## Abstract

Although numerous studies have attempted to establish the relationship between adenomyosis and infertility, no consensus has emerged. Our aim was to investigate whether adenomyosis and endometriosis affected IVF outcomes in our patients. This was a retrospective study of 1720 patients from January 2016 to December 2019. In total, 1389 cycles were included: 229 cycles in the endometriosis group (group E), 89 cycles in the adenomyosis group (group A), 69 cycles in the endometriosis and adenomyosis group (group EA), and 1002 cycles in the control group (group C). Most patients in groups A and EA received GnRH agonist treatment before FET. The 1st FET live birth rates (LBR) were 39.3%, 32.1%, 25% and 48.1% in groups E, A, EA, and C. The miscarriage rates were 19.9%, 34.7%, 39%, and 17.6%. The per retrieval cycle cumulative live birth rates (cLBRs) in patients < 38 y/o were 56.4%, 58.1%, 44.8%, and 63%. The per retrieval cycle cLBRs in patients ≥ 38 y/o were 25%, 9.8%, 17.2%, and 29.5%. Among groups A and EA, LBRs were 25.58% and 18.89% in patients with a ≥ sevenfold decrease and a < sevenfold decrease in CA-125 level, respectively, after GnRH agonist treatment. Endometriosis was not associated with a poorer pregnancy outcome. Patients with adenomyosis with/without endometriosis had higher miscarriage rates, lower LBRs, and lower cLBRs, especially in patients aged ≥ 38 years, even after GnRH agonist treatment before FET cycles. Patients who have a greater than sevenfold decrease in CA-125 level after GnRH agonist treatment might have better clinical pregnancy outcomes.

## Introduction

Adenomyosis is usually defined as the occurrence of ectopic rests of endometrium-like tissue and fibrosis resulting in an enlarged uterus. The endometrium resides deeply within the myometrium with both glands and stroma. For classification, adenomyosis can be simply divided into diffuse adenomyosis and focal adenomyosis. The development of adenomyosis is believed to involve downward invagination of the endometrial basalis layer into the myometrium and is associated with higher estrogen level and aromatase expression^1^. Symptoms are noticed in around one third of patients with adenomyosis. The prevalence of adenomyosis from hysterectomy in general population is around 20% and about the same prevalence is found by transvaginal sonography (TVS)^2,3^. TVS and magnetic resonance imaging (MRI) obtain equivalent results in the diagnosis of adenomyosis^4,5^.

Treatment of adenomyosis includes cyclic NSAID, combined oral contraceptive pills, progestin-only regimens, selective progesterone receptor modulators (SPRMs), levonorgestrel-releasing intrauterine system (LNG-IUS) and GnRH (gonadotropin-releasing hormone) agonists^6–8^. It has been shown that GnRH agonists may help women achieve a better in vitro fertilization (IVF) outcome^9^.

In infertile women, around 24% of women are diagnosed with adenomyosis, especially in those suffering from recurrent implantation failures and recurrent miscarriages, as well as in women diagnosed with endometriosis or in their last decade of reproductive age^10,11^. There are several hypothetical mechanisms in adenomyosis-associated infertility, including dysregulations of the myometrial architecture and function, chronic inflammation, presence of local oxygen, and altered endometrial function, which can cause implantation failure^12,13^. Although numerous studies have attempted to explain the relationship between adenomyosis and infertility, there are still no definitive conclusions^11,14^. Recent studies have shown adenomyosis was related to a lower clinical pregnancy rate (CPR), ongoing pregnancy rate (OGR), live birth rate (LBR), and higher miscarriage rate, regardless of maternal age and genetic status of embryos after ultra-long down-regulation GnRH agonist treatment^11,15^. However, some studies showed that adenomyosis did not affect pregnancy outcome^9,16,17^.

Endometriosis is found in around 40% of infertile women. Although it might affect natural conception rates, it is now believed that ART could bypass the toxic pelvic environment and achieve favorable clinical pregnancy rates^18,19^. In daily practice, we often notice endometriosis coexisting with adenomyosis. Moreover, there are studies reporting lower pregnancy rates in patients with endometriosis combined with adenomyosis as compared with endometriosis only^11,20^.

As mentioned above, the effect of adenomyosis and/or endometriosis on pregnancy outcome remains controversial. In this retrospective study, we analyzed patients with endometriosis, adenomyosis, or both, who underwent IVF or intracytoplasmic sperm injection (ICSI). We attempted to determine whether adenomyosis and endometriosis affected IVF outcomes in our patients.

## Materials and methods

### Subjects

This was a retrospective study of patients who underwent IVF in Taichung Veterans General Hospital, Taiwan between January 1, 2016, and December 31, 2019. It is standard practice for all of our patients undergoing IVF to receive TVS during their first visit to our hospital. Patients with a diagnosis of endometriosis and/or adenomyosis were included as the potential study group, and patients without endometriosis and/or adenomyosis or other uterine/systemic disorders were categorized as the potential control group. The inclusion criteria for the study were as follows: (1) patients underwent a cycle of IVF or ICSI, (2) endometriosis was confirmed by surgery, sonography examination, or pelvic examination, (3) adenomyosis was diagnosed by two-dimensional TVS in the past 12 months. No restriction was made for the number of previous failed cycles. The exclusion criteria were as follows: (1) patients with other uterine myoma or other anomalies, (2) patients with immune diseases or metabolic diseases, (3) patients receiving donated oocytes (4) patients receiving Preimplantation Genetic Screening (PGS).

We analyzed those patients in the study and control group who had finished their treatment cycles, either as a result of achieving a live birth or ultimately failing after transferring all of their embryos. The cumulative live birth rates (cLBRs) and cumulative ongoing pregnancy rates (cOGRs) per started cycle were followed until May 31, 2021. In order to simplify the data analysis, transfer cycles with embryos from different cycles were excluded from the study.

### Adenomyosis status

The criteria of adenomyosis include two of the five sonographic features on TVS: (1) No distinction of the endometrial–myometrial junction; (2) asymmetry of the anterior and posterior myometrium; (3) subendometrial myometrial striations; (4) myometrial cysts and fibrosis; and (5) heterogeneous myometrial echotexture. All patients received a CA-125 blood test, except for those who had already received a test previously at our hospital. For those whose CA-125 value exceeded the normal range (35 U/ml), a pre-embryo transfer (ET) GnRH agonist treatment was prescribed. GnRH agonist was prescribed at intervals of 28–35 days until the CA-125 was in normal range. The patients would undergo a CA-125 test prior to each embryo transfer. If CA-125 levels exceed the normal range, the patient would receive repeated GnRH agonist treatment.

### Controlled ovarian hyperstimulation protocol

Patients in the GnRH agonist group received leuprolide acetate (Leuprolide, 0.5 mg/day; Takeda Co., Taiwan) consisting of a daily low dose of GnRH agonist, subcutaneously administered for at least 10 days before the onset of ovarian stimulation. Meanwhile, participants in the antagonist group received the GnRH antagonist cetrorelix acetate (Cetrotide, 0.25 mg/day SC; Merck Serono, German) starting flexibly on stimulation days 5–7 by ultrasound monitoring 5 days after the onset of COH with gonadotropins.

The types and dosages of gonadotropin administration were individualized for each participant according to her age, body mass index, anti-mullerian hormone level (AMH), FSH/LH level, antral follicle counts on cycle day 2–3 and response to previous ovarian stimulation. Doses were adjusted according to the ovarian response as monitored by vaginal ultrasound folliculometry and serum E2 level testing.

When two or more follicles reached a mean diameter of 18 mm, 10,000 IU of hCG (Pregnyl, Merck Sharp & Dohme, America; Organon) or 250–500 µg of recombinant hCG (Ovidrel; Merck Serono, German) was injected for the oocyte retrieval 35–36 h later.

For the frozen-thaw cycle, patients received an artificial hormone replacement regimen (estradiol valerate 2 mg, Synmosa Co., Taiwan) with a step-up from 4 to 8 mg per day for 5 days, to 6–12 mg per day for 5 days, followed by twice daily vaginal progesterone (8% Crinone; Merck Serono, German) 90 mg plus estradiol valerate 12 mg once endometrial thickness exceeded 8 mm on ultrasound assessment. Then the dosage was maintained till the patient reached 10 complete weeks of gestation for luteal support (LS).

In addition, 0.1 mg Decapeptyl (Ferring Co., Germany) was also administered on the 6th day after ICSI as a measure of adjuvant LS. The embryo transfers (ET) were carried out on day 2, day 3, or day 5 of culture.

### Outcome measures

Serum βhCG was checked on embryonic age day 14, and measurements over 5 mIU/mL were defined as a biochemical pregnancy if no gestational sac could be identified later.

Clinical pregnancy was defined as the presence of a gestational sac under TVS at the gestational age of 4–5 weeks. Ongoing pregnancy was defined as a pregnancy that had completed ≥ 20 weeks of gestation. Live birth rate was defined as the number of deliveries that resulted in a live born neonate.

Cumulative live birth rates (cLBR) per retrieval cycle were defined as the percentage of at least one live born neonate from that retrieval cycle. Patients with embryos transferred from mixed cycles were excluded in cLBR. The above results were followed until May 31, 2021.

### Statistical analyses

Statistics were conducted by SPSS-PC ver. 22.0. Patient’s characteristics, AMH, oocyte retrieval number, and pregnancy outcomes were analyzed using the two-tailed t test and Chi-squared test among groups, with a p value of less than 0.05 considered statistically significant.

### Ethical approval

The study protocol was approved by the Institutional Review Board of Taichung Veterans General Hospital on December 1, 2021 with approval code CE2147, and adhered to relevant ethical guidelines. Human participants' names and other HIPAA identifiers had been removed from all sections of the manuscript and data. The Institutional Review Board of Taichung Veterans General Hospital approved to waive the documentation of informed consent due to this research presented no more than minimal risk of harm to subjects which involves only data review.

## Results

### Patient’s characteristics

In total, 1720 cycles were reviewed and analyzed in the study period. There were 1179 women and 1389 cycles enrolled in the study using inclusion and exclusion criteria. The cycles were grouped as follows: 229 cycles in the endometriosis group (group E), 89 cycles in the adenomyosis group (group A), 69 cycles in the endometriosis and adenomyosis group (group EA), and 1002 cycles in the control group (group C). Baseline demographics, cycle characteristics, and laboratory culture results of the four groups are summarized in Table 1.

**Table 1:** Baseline demographics, cycle characteristics and culture results of the four groups.

Parameters	Endometriosis (group E) (n = 229)	Adenomyosis (group A) (n = 89)	Endometriosis + adenomyosis (group EA) (n = 69)	Control (group C) (n = 1002)
Age (years)	34.8 ± 4	38 ± 3.7	37.6 ± 4.3	37 ± 4.2
BMI (kg/m^2^)	21.7 ± 2.9	22.4 ± 2.9	22.5 ± 3.2	22.7 ± 3.5
AMH (ng/ml)				
Average	2.6 ± 2.2	1.8 ± 2.0	2.0 ± 1.7	2.9 ± 3.1
< 38 (year-old)	2.7 ± 2.1	2.6 ± 2.2	2.2 ± 1.7	3.6 ± 1.7
≥ 38 (year-old)	1.8 ± 2.2	1.3 ± 1.7	1.8 ± 1.7	2.0 ± 2.3
Rates of primary/secondary infertility	64.6%/35.4%	51.7%/48.3%	73.9%/26.1%	54.7%/45.3%
Induction duration (days)	10.0 ± 2.0	9.8 ± 2.0	10.0 ± 2.0	9.9 ± 1.9
Total dosage of FSH (IU)	3595.13 ± 2158.7	3639 ± 2271.4	3421.4 ± 1728.8	3612 ± 2272.8
Total dosage of LH (IU)	1090.3 ± 658	1126 ± 563	1343.5 ± 632.5	1086.5 ± 614.6
Estradiol on day of hCG administration (pg/mL)	2131.5 ± 1534.9	1538.1 ± 1543.5	1801.5 ± 1697.7	2322.2 ± 1942.5
Progesterone on day of hCG administration (ng/mL)	0.9 ± 1.4	0.7 ± 0.5	0.7 ± 0.4	0.8 ± 0.5
No. of oocyte retrieved	10.3 ± 7.8	7.8 ± 7.6	6.4 ± 5.0	11.2 ± 9.2
No. of mature oocytes	8.0 ± 6.3	5.6 ± 6.4	5.3 ± 4.6	8.7 ± 7.3
No. of oocytes fertilized	7.6 ± 6.3	5.4 ± 5.5	5.2 ± 4.3	7.7 ± 6.8
Fertilization rates	72.1%	70.6%	77.9%	69.4%
Rates of good embryos^a^ (at day 3)	28%	28.6%	31.8%	32.3%
Rates of good blastocyst^b^ (at day 5/6)	30.4%	28.8%	26.4%	33.67%
Rates of blastocyte for cleavage (from cleavage stage embryos)	53.7%	50.7%	45.8%	52.4%
Rates of cleavage stage/blastocyst stage transfer at 1st FET	41%/59%	41%/59%	36%/64%	48%/52%

^a^Cleavage embryos were defined as good quality embryo if they were composed of at least seven-to-eight cell grade 1 or 2 on day 3, according to the Veeck classification system.

^b^Grade according to Gardner classification system of “3BA” or greater was defined as good quality blastocyst.

The average value of CA-125 before GnRH agonist treatment was 104.8 U/ml in group A and 167.9 U/ml in group EA. The average CA-125 value after treatment was 22.0 U/ml in group A and 26.5 U/ml in group EA before starting hormonal replacement therapy in preparation for embryo transfer. A total of 72 cycles (80.9%) in group A and 54 cycles (85.6%) in group EA received GnRH agonist treatment before embryo transfer.

The LBRs of 1st frozen-thawed embryo transfer cycle (FET) were significantly lower in group A and group AE as compared to those in group E and group C (Table 2). Subgroup analysis also revealed significant differences between groups E and C, and groups EA and C in patients younger than age 38 years. Significantly lower LBRs were also noted in group A compared with group C, and in group EA compared with group C in patients older than age 38 years. Our findings suggest that patients with co-occurring endometriosis and adenomyosis exhibit a significantly lower clinical pregnancy rate as compared to patients with either condition alone. Table 2 also showed patients with adenomyosis had a higher tendency towards miscarriage rate and led to a lower live birth rate.

**Table 2:** Pregnancy outcomes of 1st frozen-thawed embryo transfer cycles (FET) of the four groups.

Pregnancy outcome	Endometriosis (group E) (n = 229)	Adenomyosis (group A) (n = 89)	Endometriosis + adenomyosis (group EA) (n = 69)	Control (group C) (n = 1002)	P value
CPR in 1st FET cycles	49.1%	49.1%	41%	58.4%	EA vs C 0.029 Other: NS
LBR in 1st FET cycles	39.3%	32.1%	25.0%	48.1%	EA vs C 0.003 A vs C 0.028 Other: NS
Clinical miscarriage rates	19.9%	34.7%	39%	17.6%	NS

*NS* non-significant P-values.

The cumulative pregnancy outcomes per retrieval are presented in Table 3. There were significantly lower cLBRs in groups A and EA compared with group E and C. In the sub-group analyses according to age, the lower average cLBR in groups A and EA was mostly due to older patients (age ≥ 38 years). The abortion rates were higher in group A and AE without significant differences. The results suggest that adenomyosis has a greater impact on older patients as compared to younger patients.

**Table 3:** Cumulative live birth rates per retrieval cycle of the four groups.

Pregnancy outcome	Endometriosis (group E) (n = 208)	Adenomyosis (group A) (n = 72)	Endometriosis + adenomyosis (group EA) (n = 58)	Control (group C) (n = 957)	P value
Per retrieval cycle	48.6%	30.6%	31.0%	52.9%	E vs A 0.036 E vs EA 0.028 A vs C 0.025 EA vs C 0.02 Other: NS
Per retrieval cycle, with patients age < 38	56.4%	58.1%	44.8%	63%	EA vs C 0.049 Other: NS
Per retrieval cycle, with patients age ≥ 38	25.0%	9.8%	17.2%	29.5%	A vs C 0.007 Other: NS

*NS* non-significant P-values.

Patients with embryos transferred from mixed cycles were excluded.

Table 4 shows the subgroup analysis of the clinical outcomes in the 1st FET cycles by the initial serum CA-125 and fold decrease in CA-125 level after treatment in group A and group EA. There were no significant differences in CPR and LBR among the different CA-125 levels, including cut-off values set at 100, 150, or 200 U/ml. The analysis of fold decrease in CA-125 level after treatment revealed that patients who had a greater than sevenfold decrease had a significantly higher CPR.

**Table 4:** The clinical outcomes of FET cycles by the initial serum CA-125 and fold decreases in CA-125 after treatment in group A and group EA.

	Initial CA-125 ≥ 100 U/ml	Initial CA-125 < 100 U/ml	P value
Clinical pregnancy rates	35.71% (25/70)	31.17% (24/77)	0.678
Live birth rates	24.29% (17/70)	22.08% (17/77)	0.853

	CA-125 decrease folds ≥ 7 times	CA-125 decrease folds < 7 times	P value
Clinical pregnancy rates	44.19% (19/43)	26.67% (24/90)	0.043*
Live birth rates	25.58% (11/43)	18.89% (17/90)	0.376

*p < 0.05.

On May 31, 2021, there were still 4 patients in group E, 1 patient in group A, and 12 patients in group C with an ongoing pregnancy who are not included in this study. The follow up before publication showed 1 termination due to fetal anomalies in group A and 1 fetal death in group C.

## Discussion

To the best of our knowledge, no studies have been conducted to determine the cumulative live birth rate of patients with endometriosis and adenomyosis with and without endometriosis under GnRH agonist pre-treatment before FET. Our study compared the effects of these diseases on their respective pregnancy outcomes. We evaluated the live birth rates after first transfer of frozen thawed embryos and the final cumulative live birth rates based on per retrieval cycle basis, with a further sub-analysis of the patients by age. These data showed clearly that in patients with adenomyosis undergoing GnRH agonist pretreatment before FET, cumulative LBRs were not inferior in patients aged < 38 years but were significantly lower in those aged ≥ 38 years, compared with the control and endometriosis groups. Moreover, the endometriosis group achieved a cumulative LBR comparable with that of the control group.

There was no clear evidence indicating whether or not adenomyosis impacted the clinical pregnancy rates, and its effect on cumulative pregnancy rates was even less clear. It remains debatable as to what strategy should be adopted for embryo transfer in adenomyosis patients. In Table 5, we have summarized the available studies in the literature which have investigated the impact of adenomyosis and/or endometriosis on pregnancy outcome by IVF/ICSI. Three studies found that adenomyosis had no impact on pregnancy rates^9,16,17^. Other studies reported adverse influences of adenomyosis on pregnancy rates^10,11,15,21–23^. In studies with ultralong GnRH agonist protocol before fresh ET, the results found conflicting conclusions^9,10,24^.

**Table 5:** Main characteristics of published studies on adenomyosis, endometriosis, and pregnancy outcomes.

Study design	Number of patients		Treatment protocols and IVF characteristics	CPR		LBR		Authors/year
	Adenomyosis	Control		Adenomyosis	Control	Adenomyosis	Control	
Retrospective cohort study	20	54	Long-term (> 3 months) GnRH agonist prior to first IVF/ICSI cycle			35%	30%	Mijatovic et al., 2010^9^
Retrospective cohort study	37	164	Only fresh ET	35%	31%	29.7% (OGR)	29.3% (OGR)	Costello et al., 2011^16^
Retrospective cohort study	38	137	Only D4/5 fresh ET	23.6%	43.6%			Thalluri et al., 2012^23^
Prospective multicenter	49	49	Only asymptomatic Adenomyosis patients with fresh ET	43%	29%	35%	18%	Benaglia et al., 2014^17^
Retrospective cohort study	34	137	All patients underwent PGS, 79.4% (n = 27) adenomyosis underwent ultra-long GnRH-agonist down regulation			47%	80%	Stanekova et al., 2018^15^
Prospective multicenter cohort study	120 (fresh) 79 (FET)	335 (fresh) 170 (FET)		23.1% (fresh) 40.5% (FET)	35.6% (fresh) 40.0% (FET)	11.5% (fresh) 24.1% (FET)	17.0% (fresh) 23.5% (FET)	Higgins et al., 2021^22^
Retrospective matched cohort study	Adenomyosis/endometriosis/ control: 328/242/331		All received oocytes donation	Adenomyosis/endometriosis/ control: 40%/44%/30.8%		Adenomyosis/endometriosis/ control: 26.8%/38%/37.1%		Martinez-Conejero et al., 2011^21^
Retrospective study	Adenomyosis: Fresh ET without GnRH-a pretreatment/ Fresh ET with GnRH-a pretreatment /FET with GnRH-a Pretreatment: 147/105/43			Fresh ET without GnRH-a pretreatment/ Fresh ET with GnRH-a pretreatment /FET with GnRH-a Pretreatment: 25.2%/30.5%/39.5%				Park et al., 2016^10^
Retrospective cohort study	Adenomyosis/endometriosis/adenomyosis + endometriosis/control: 64/88/355/466		Adenomyosis group underwent ultra-long down regulation, All D2 or D3 ET	Adenomyosis/endometriosis/ adenomyosis + endometriosis/control: 23.44%/36.55%/22.72%/35.55%		Adenomyosis/endometriosis/adenomyosis + endometriosis/control: 12.5%/26.48%/11.36%/27.47%		Sharma et al., 2019^11^
Retrospective cohort study	Adenomyosis FET pretreatment/without pretreatment: 48/140 Cumulative LBR group Pretreatment/without pretreatment: 97/216		All adenomyosis patients, GnRH agonist pretreatment at early follicle phase up to 3 doses			Adenomyosis FET pretreatment/without pretreatment: 37.7%/21.2% Cumulative LBR group Pretreatment/without pretreatment: 40.5%/27.9%		Chen, M et al., 2020^24^

In a study reporting that adenomyosis has a negative effect on pregnancy outcomes^16^ overdiagnosis of adenomyosis was noted as a concern. However, this hypothesis was refuted in a study conducted the following year^23^. An investigation^9^ of patients receiving GnRH-agonist treatment reported that adenomyosis had no effect on LBR. In the aforementioned study longer GnRH-agonist treatment (average 5 months) was prescribed compared with our study (2.2 months in group A and 2.5 months in group EA). The pregnancy outcomes of the adenomyosis group in two prior studies^9,16^ were similar to our results (LBR 35%, 29.7%, our study 32.1%). However, the pregnancy outcomes of the control group were both lower than those in our study, which might be the key reason that led to the different conclusions (LBR 30%, 29.3%, our study 48.1%). Although a recent study^22^ reported CRP and LBR in adenomyosis group that were not inferior, the results also showed that FET was related to better pregnancy outcomes compared with fresh ET. FET seemed to be a better option and was also adopted in our study for groups A and EA. However, the aforementioned study showed lower pregnancy outcomes in both control and adenomyosis groups compared with our results. In three prior studies^9,16,22^, there were no pretreatment CA-125 data available for further comparisons between baseline and changes in the disease severity. In our data, there were no differences in first ET, i.e., fresh ET in groups E and C compared with 1st frozen ET in groups A and EA were not significantly different. However, FET in groups E and C showed significant improvement.

For studies showing a negative impact of adenomyosis on pregnancy rates, a 2012 study revealed a similar pregnancy outcome to that found in our study^23^. A retrospective study with a relatively large number of patients revealed a decrease of CPR and LBR in the adenomyosis group under ultra-long GnRH agonist treatment^11^. Although the aforementioned study and our study have similar conclusions, our patients were treated with a different treatment strategy and displayed better pregnancy outcomes (LBR 32% vs 11.36%) in a relatively older patient population.

Our results support the hypothesis that adenomyosis does adversely impact pregnancy outcomes. Our data showed that in patients aged < 38 years, cLBRs per retrieval cycle increased around 5–10% per cycle compared with the results of their first ET. Nevertheless, in patients aged ≥ 38 years, the cLBRs per retrieval cycle in both group A and EA were less than 20%. The lower cLBRs in adenomyosis patients could mostly be explained by patients being aged ≥ 38 years. Adenomyosis probably irreversibly injures the myometrial architecture and function after years of disease progression^13^. If the damage is severe enough, it might still affect implantation potential even after applying GnRH agonist pre-treatment to achieve a seemingly normal CA-125 value, eventually leading to a lower chance of achieving a live birth chance.

Previous studies showed that the impacts of endometriosis on fertility can mostly be overcome by IVF^18,19,25^. ART could achieve pregnancy outcomes that are relatively close to those attained in patients with male factor^19^. Our study found the same results, namely, that endometriosis had no negative effects on LBRs in fresh ET, FET, and cLBR per retrieval cycle compared with the control group. Our study findings also suggest that the outcomes of adenomyosis patients were also diagnosed with endometriosis were similar to those of adenomyosis patients.

Whether the poor pregnancy outcomes in patients with adenomyosis are due to decreased implantation rates or increased miscarriage rates has not been clearly established^26^. There is a higher spontaneous abortion rate in patients with adenomyosis, which may require enhanced luteal support^27^. In another study, it was reported that donated oocyte cycles showed a miscarriage rate twice that of the endometriosis and control groups^21^. Furthermore, patients receiving PGT-A showed a threefold greater rate of miscarriage in the adenomyosis group^15^. In addition, an investigation of GnRH agonist treatment reported decreased live birth rates and increased miscarriage rates^11^. Our study revealed a higher miscarriage rate in adenomyosis patients. The miscarriage rates were similar between our data and the findings of the abovementioned study^11^. More studies are needed to establish whether there is an impact on CPR and, furthermore, to explore the effects in patients under different treatment strategies.

GnRH agonist is believed to reduce inflammatory reaction, mitigate the angiogenic response, and induce apoptosis in tissue from patients with adenomyosis^28^. It was reported that among 3 groups (fresh ET with/without GnRH agonist treatment before cycles, GnRH agonist treatment before FET cycles), GnRH agonist treatment before FET cycles showed the best clinical pregnancy rates, compared with fresh ET both with and without GnRH pretreatment^10^. Moreover, it has also been demonstrated that FET cycles with long-term GnRH agonist pretreatment significantly improved pregnancy outcomes^29,30^. We use GnRH agonist pretreatment before FET as our ET strategy in most of our adenomyosis patients and monitored CA-125 levels to guide the length of treatment duration. Our results showed that under GnRH agonist pretreatment before FET to achieve a relatively normal CA-125 serum level, the live birth rates of patients with adenomyosis approached those of patients under the age of 38 years in groups E and C. However, in patients aged ≥ 38 years, LBRs and cLBR were still lower than those of the control group.

Our subgroup analysis showed that serum CA-125 levels before GnRH agonist treatment were not associated with CPR or LBR at cut-off values of 200, 150, and 100 U/ml. We demonstrated that patients could achieve non inferior pregnancy outcomes after GnRH agonist treatment before FET, even with a high CA-125 level. However, there was no LB in patients with pretreatment CA-125 > 350 U/ml. The subgroup analysis of the fold decrease in CA-125 level after treatment revealed that patients with a sevenfold decrease had significantly higher CPRs.

Our study has notable strengths. This is the first study to compare the impact of endometriosis and adenomyosis in patients receiving GnRH pre-treatment on cLBR and to include age in the analysis. The limitation of this retrospective study was that we did not enroll group of adenomyosis patients without GnRH agonist treatment. The diagnosis of adenomyosis and endometriosis was not based on surgical findings. Due to the lack of fresh ET data, we did not analyze the impact of adenomyosis on the pregnancy outcome between fresh ET and FET. There are also no data on the change in uterus volume after GnRH agonist treatment due to incomplete data. We might have missed some patients with endometriosis in adenomyosis group due to not all patients received laparoscopies.

In conclusion, endometriosis was not associated with a poorer pregnancy outcome. Patients with adenomyosis, or both adenomyosis and endometriosis, have a higher miscarriage rate, lower LBRs, and lower cLBRs, especially in patients aged ≥ 38 years even after GnRH agonist treatment before FET cycles. Patients who have a greater than sevenfold decrease in CA-125 level after GnRH agonist treatment might have better clinical pregnancy outcomes.

## Data Availability

The datasets used and analyzed during the current study available from the corresponding author on reasonable request.
